# Small Bowel Ischemia due to Jejunum Volvulus in Pregnancy: A Case Report

**DOI:** 10.1155/2012/485863

**Published:** 2012-12-12

**Authors:** Ioannis Vassiliou, Aliki Tympa, Michalis Derpapas, Georgios Kottis, Nikolaos Vlahos

**Affiliations:** ^1^Second Department of Surgery, Aretaieion University Hospital, 76 Vas. Sofias Avenue, 11528 Athens, Greece; ^2^First Department of Anesthesiology, Aretaieion University Hospital, 76 Vas. Sofias Avenue, 11528 Athens, Greece; ^3^First Department of Obstetrics and Gynecology, Aretaieion University Hospital, 76 Vas. Sofias Avenue, 11528 Athens, Greece

## Abstract

The diagnosis of intestinal obstruction in pregnancy is difficult, as the symptoms may mimic pregnancy-associated complaints. The surgical management is challenging, as the mortality rate of midgut volvulus in pregnancy is high. We report the case of a 35-year-old woman at 21 weeks and 5 days of gestation with small bowel obstruction who presented to our institution with a 24 h history of colicky abdominal pain and nausea and who finally had a successful open repair.

## 1. Introduction

Volvulus is the second most common cause of intestinal obstruction in pregnancy, occurring in up to 25% of the cases as compared to only 3–5% in nonpregnant women. The diagnosis of intestinal obstruction in pregnancy is difficult, as the symptoms may mimic pregnancy-associated complaints. The abdominal distention due to uterine enlargement and the displacement of the viscera from the gravid uterus may alter the typical signs of the acute abdomen. The surgical management is challenging, as the mortality rate of midgut volvulus in pregnancy is high.

In this paper, we report the case of a 35-year-old woman at 21 weeks and 5 days of gestation with small bowel obstruction who presented to our institution with a 24 h history of colicky abdominal pain located at the epigastrium and nausea and who finally had a successful open repair.

## 2. Case Presentation

A 35-year-old woman at 21 weeks and 5 days of gestation presented to our institution with a 24 h history of colicky abdominal pain located at the epigastrium and nausea. She also reported constipation for the past 3 days with only one bowel movement. On admission, her vitals signs were stable. Clinical examination revealed generalized mild abdominal tenderness, with local guarding to the left abdomen. Rebound tenderness was not present at the time. Mild bowel sounds were present with high frequency and there was no uterine tenderness. Rectal examination revealed an empty rectum. Evaluation of the pregnancy did not show uterine contractions. Laboratory evaluation on admission showed WBC of 8.8.000/mL with 69% poly and Ht of 36%. Her surgical history included an uncomplicated open excision of a benign mesenteric cyst, eight years ago and a cesarean section, two years ago. During the course of her hospitalization, however, her condition gradually deteriorated. Her repeat blood count revealed leukocytosis (18,500/mm^3^) with left shift (90% poly) and elevated C-reactive protein (CRP) of 11.1 mg/dL. Cardiotocogram was normal. Transabdominal ultrasound (TUS) revealed dilated small bowel loops and free fluid in the Douglas pouch. Magnetic resonance imaging (MRI) of the abdomen confirmed the diagnosis of small bowel obstruction.

**Figure 1:**
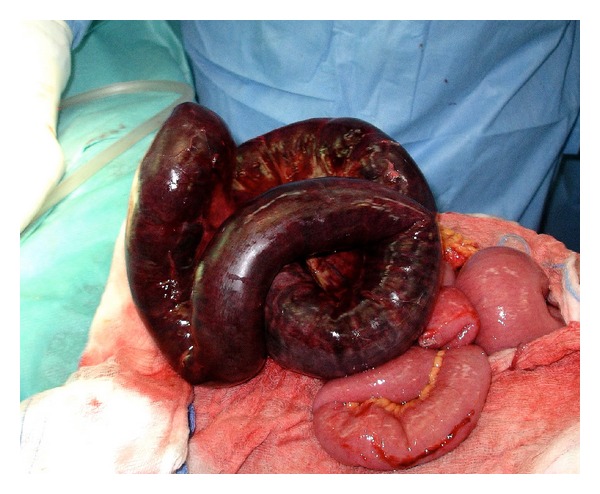
The jejunum is dark and edematous, indicating ischemia.

The patient underwent immediate exploratory laparotomy through a midline incision. Upon entering the abdominal cavity, a moderate amount of bloody peritoneal fluid was noted. Peritoneal fluid cultures were obtained. Volvulus of the jejunum was present, around the mesentery, 60 cm from the Treitz ligament. The bowel was dark and edematous indicating gangrenous of the jejunum (Figure 1). Although there was a foul smell, no bowel perforation was noted. The necrotized segment was excised and isoperistaltic handmade side-to-side jejuno-jejunostomy was performed, in two layers with PDS no. 4-0. The decision for such an anastomosis was made because the proximal jejunum was edematous and 3 times dilated with respect to the distal jejunum. A thorough peritoneal lavage was performed and the relevant antibiotics were administered intra- and postoperatively. The patient had an uneventful postoperative course. The well-being of the pregnancy was also confirmed three weeks later. The patient underwent an uncomplicated pregnancy until 39 weeks of gestation when she underwent elective cesarean delivery of a liveborn healthy male neonate.

## 3. Discussion

Volvulus is the second most common cause of intestinal obstruction in pregnancy, occurring in up to 25% of the cases as compared to only 3–5% in nonpregnant women. Other causes include intussusception, hernia, and cancer [1]. Furthermore, the mortality rate of midgut volvulus in pregnancy is significantly higher (3–15%) with respect to the general population [1–3].

Recently, Unal et al. presented a 20-case series of acute abdomen in pregnancy. US findings agreed with surgical findings in only 55%, while MRI was successful in 83% [2]. Previous abdominal surgery may predispose to bowel obstruction in pregnancy.

The diagnosis of intestinal obstruction in pregnancy is difficult, as the symptoms may mimic pregnancy-associated complaints. The abdominal distention due to uterine enlargement and the displacement of the viscera from the gravid uterus may alter the typical signs of the acute abdomen.

For the above-mentioned reasons, the diagnosis of bowel obstruction in pregnancy might be overlooked. MRI may offer significant advantages in such cases without the risk of ionizing radiation [4].

In pregnancy regardless of imaging results, when bowel obstruction is highly suspected, an exploratory laparotomy should be performed immediately. The entire bowel should be searched for obstruction points. Intestinal obstruction may be complicated by peritonitis, with severe consequences and preterm labor. As intra-abdominal surgery can also result in premature uterine contractions, tocolytic agents can be used preventively before surgery and during the immediate postoperative course.
